# Factors associated with the rapid implementation process of the fixed-dose combination RHZE tuberculosis regimen in brazil: an ecological study

**DOI:** 10.1186/1471-2458-13-321

**Published:** 2013-04-09

**Authors:** José Ueleres Braga, Deborah Araújo da Conceição, Anete Trajman

**Affiliations:** 1 Professor Helio Fraga Reference Center, National School of Public Health, Rio de Janeiro, Fiocruz, Brazil; 2 Social Medicine Institute, Rio de Janeiro State University, Rio de Janeiro, Brazil; 3 Health Education Post-graduation Program, Gama Filho University, Rio de Janeiro, Brazil; 4 Montreal Chest Institute, McGill University, Montreal, Canada

**Keywords:** Tuberculosis, Drug combinations, Health plan implementation, Epidemiology, Public health

## Abstract

**Background:**

The Brazilian National Tuberculosis Control Program (NTCP) recommended the fixed-dose four-drug combination (FDC-RHZE) regimen to treat new tuberculosis cases in December 2009, expecting to increase adherence and avoid resistance. We evaluated factors associated with the speed of the new regimen implementation process in this continent-sized country.

**Methods:**

We conducted an ecological study based on the Brazilian Case Notification Database (SINAN) having the Brazilian municipalities as the analytical unit. Municipalities with at least one case reported from December 2009 to March 2011 were considered eligible. The association of rapid (≤ 3 months) implementation of the new regimen with demographic, epidemiological and operational health service characteristics, such as compliance with NTCP recommendations (supervised treatment, bacteriological confirmation of the diagnosis and monthly bacteriological monitoring), was analyzed. We used the adjusted odds ratios (OR) and their 95% confidence interval (CI) to assess the association of independent variables with the outcome in a multiple logistic regression model.

**Results:**

Rapid implementation of the new regimen in municipalities was associated with small populations (OR=25.5, 95% CI= 19.1-34.1), low population density (OR=2.3, 95% CI= 1.9–2.9), low tuberculosis incidence rates (OR=8.8, 95% CI= 6.7–11.4) and good compliance with other NTCP recommendations.

**Conclusions:**

We showed that SINAN secondary data analysis is feasible and useful to learn lessons from. Municipalities with high tuberculosis burden and large populations need special attention for implementing new recommendations. This is particularly important considering the Global Alliance pipeline for new tuberculosis treatment regimens.

## Background

In 1996, the Brazilian National Tuberculosis Control Program (NTCP) incorporated the directly observed treatment strategy (DOTS) for reaching World Health Organization (WHO) cure targets and reducing default rates, thus preventing emergence of resistant bacillus [1]. In the following years, as in other countries that recommended DOTS, a decrease in default rates and an increment in case detection and cure rates were observed in Brazil. Nevertheless, this was insufficient to achieve the target for tuberculosis control in the country [2,3].

By the end of 2009, Brazil was the only high-burden country to use a treatment regimen with only three drugs [fixed-dose combination (FDC)-Rifampin and Isoniazid (RH) and Pyrazinamide (Z)]. Despite a free-of-charge treatment, the default rates were around 9.3%, attaining 14% in some states [4]. At this point, the NTCP advisory board revised the Brazilian Tuberculosis Guidelines and recommended the WHO-suggested four-drug FDC regimen for tuberculosis treatment for adolescents and adults (over 10 years old). Besides including Ethambutol (E), the new regimen reduced doses of H (from 400 to 300 mg) and of Z (from 2000 to 1600 mg). The new FDC-RHZE regimen was expected to result in lower default rates and higher effectiveness of treatment. The rational was that the smaller number of pills to be taken and the better tolerance due to dose reduction would increase adherence to treatment [5]. Finally, the NTCP expected that the inclusion of a fourth drug would fight the emergence of resistance, which was, at that point, widely spread in Eastern Europe, Asia and Africa [6].

According to the Brazilian Ministry of Health (MoH), the implementation of the FDC-RHZE regimen has followed a schedule set by the NTCP in agreement with the Brazilian states’ programs [5]. The use of this regimen started in the North (Amazonian) region, which had the highest incidence rate until the 90’s, while the Southeast region, presently with the highest disease burden, was the last one to implement the new regimen [7]. The effective use of the new regimen may have faced important barriers in healthcare services, such as poor adhesion by professionals and by tuberculosis patients.

New tuberculosis treatment regimens and diagnostic technologies in the health system are expected during the following years [8-12]. Understanding the barriers and bottlenecks that could delay the incorporation of these technologies may be useful for the NTCP stakeholders. In Brazil, several public health programs [8-12], including DOTS [13], had their implementation process investigated. However, no published studies on the implementation process of the recently adopted therapeutic regimen for tuberculosis were found in the literature.

The objective of the present study is to investigate socio-demographic, epidemiological and operational factors associated with the implementation process of the FDC-RHZE regimen for tuberculosis treatment in Brazilian municipalities. The understanding of this process may identify areas with poor adhesion to the new intervention and eventually guide the MoH in future implementation of health programs and policies in the country.

## Methods

The analysis unit of this ecological study is the Brazilian municipalities. Data from the Brazilian Case Notification Database (SINAN-Tuberculosis) were used. All municipalities with at least one tuberculosis case notified at SINAN were considered eligible. Population size estimates were those calculated by the Brazilian Geography and Statistics Institute (IBGE) and are available on the Informatics Department of the Brazilian National Health System’s website (http://www.datasus.gov.br). Patient records from SINAN database include which drugs were used but not the regimen (i.e., whether FDC was in use). Thus, new adolescent and adult (>10 years) cases notified between December 2009 and March 2011 using the four drugs (R, H, Z and E) after the starting month of implementation of the FDC-RHZE, as informed by the State Health Secretaries, were considered as users of the new FDC-RHZE regimen. We performed an initial quality evaluation on this data frame, aiming to identify data completeness, errors and inconsistencies. We concluded that the database was adequate for the present analysis [7].

The following epidemiological and demographic municipal explanatory factors were analyzed: *i-* population size, *ii-* population density and *iii-* tuberculosis incidence (absolute number of cases and notification rate). Operational health unit explanatory factors included other NTCP recommendations [1]: *i-* use of directly observed treatment (DOT-), *ii-* bacteriological confirmation of cases (positive sputum smear or culture for *M. tuberculosis*) and *iii-* bacteriologic monitoring of treated cases (monthly sputum smear). We initially evaluated two outcomes: *i-* period of onset of the new regimen implementation and *ii-* implementation speed, i.e., the duration of time until maximum coverage (100%) of the new regimen was reached. After an initial exploratory analysis with three ranges (≤2 months, 2–4 months and ≥ 4 months), we chose to work with a 3 month cut-off. Rapid implementation was defined as up to three months (≤3 months) from the onset to maximum coverage. This period can be considered adequate for the Brazilian health system based on its decentralized structure.

Exploratory data analysis also evaluated the distribution of the socio-demographic, epidemiological and operational health service performance variables. We used the odds ratio (OR) to compare the groups of interest. We obtained crude and adjusted estimates in single and multiple regression models using the STATA 10.0 software [14]. The 95% confidence intervals (CI) was calculated using the Mantel-Hanszel method. Explanatory variables with a marginal association with the outcome (p≤0.20) in the bivariate analysis were included into the multiple regression model.

This study was approved by the Institutional Review Board of the Social Medicine Institute (process n° 0024.0.259.000-11) and by the National Committee of Ethics in Research (CONEP, process n°16776).

## Results

The municipalities’ characteristics are described in Table 1. Sixty-nine percent (3,826) of Brazilian municipalities were included. Over 70% implemented the new regimen between the 4^th^ quarter of 2009 and the 1^st^ quarter of 2010; 45% reached maximum coverage in less than two months (Table 1).

**Table 1:** Characteristics of the municipalities that implemented the FDC-RHZE regimen in Brazil

**Characteristics**	**n**	**%**
Time to achieve maximum coverage by the FDC-RHZE regimen		
Less than 2 months	1722	45.0
2 to 4 months	599	15.7
More than 4 months	1505	39.3
Period of onset of the FDC-RHZE regimen implementation		
4^th^ quarter 2009 to 1^st^ quarter 2010	2734	71.5
2^nd^ quarter 2010 to 3^rd^ quarter 2010	734	19.2
4^th^ quarter 2010 to 1^st^ quarter 2011	358	9.3
Population of municipalities		
Less than 10,000 inhabitants	1062	27.8
10,000-50,000 inhabitants	2156	56.3
50,000-250,000 inhabitants	509	13.3
250,000-500,000 inhabitants	61	1.6
More than 500,000 inhabitants	38	1.0
Classification of the population density		
Less than 11 inhabitants/km^2^	817	21.4
11-22 inhabitants/km^2^	736	19.2
22-44 inhabitants/km^2^	906	24.0
44-88 inhabitants/km^2^	609	16.0
More than 88 inhabitants/km^2^	755	19.4
Level of incidence		
Very low < 10/100,000	523	13.7
Low 10-25/100,000	1081	28.2
Intermediate 25-50/100,000	1329	34.7
High 50-100/100,000	667	17.4
Very high >100/100,000	226	6.0
Proportion of cases in DOT*		
Very low <20%	114	3.0
Low 20-40%	36	1.0
Intermediate 40-60%	55	1.4
High 60-80%	88	2.3
Very high >80%	3533	92.3
Laboratory confirmation of diagnosis		
Very low <20%	574	15.0
Low 20-40%	323	8.4
Intermediate 40-60%	672	17.5
High 60-80%	784	20.5
Very high >80%	1473	38.6
Laboratory monitoring		
Very low <20%	983	25.7
Low 20-40%	310	8.2
Intermediate 40-60%	567	14.8
High 60-80%	633	16.5
Very high >80%	1333	34.8

* DOT- Directly observed treatment.

Few municipalities (2%) have more than 250,000 inhabitants, one-fifth have a high population density (over 88 inhabitants per km^2^) and the same proportion has a density lower than 11 inhabitants per km^2^.

Most of the municipalities had a low or intermediate incidence rate. Noteworthy, more than 90% of municipalities have a very high proportion (more than 80%) of cases under DOT (Table 1).

Over half of the municipalities perform laboratory confirmation for tuberculosis in more than 60% of diagnosed cases. As for laboratory monitoring of treated cases, 34% perform in less than 40% of cases and a similar proportion in more than 80% of cases (Table 1).

Because the onset date of implementation did not have a clear trend, and no relationship was detected between the explanatory factors and the onset of the implementation (data not shown), only rapid implementation was analyzed as the outcome in the present study.

Table 2 presents demographic, epidemiological and operational performance characteristics according to the time to reach maximum coverage of the new RHZE-FDC regimen. The more populated the municipalities are, the longer it takes to reach maximum coverage. Conversely, the higher the demographic density and incidence rates, the slower the implementation. Based on this exploratory analysis, the cut-off points of the independent variables were set as shown in Table 3.

**Table 2:** Epidemiological characteristics and NTP performance according to the time to full implementation of the FDC-RHZE regimen in Brazil

**Characteristics**	**Time to full implementation of FDC-RHZE regimen in Brazil**			
	**Less than 2 months**	**2 to 4 months**	**More than 4 months**	**Total**
Demographic and epidemiological				
Size population (mean)	11756	17941	98974	47032
Population density (mean)	35.2	46.4	313.2	146.4
Tuberculosis incidence rate* 2010 (mean)	25.3	32.2	67.3	42.9
Operational NTP**				
Proportion of patients with DOT*** (mean)	96.4	95.5	92.4	94.7
Proportion of patients with confirmatory laboratory tests (mean)	60.6	62.7	64.6	62.5
Proportion of patients with follow-up laboratory tests (mean)	49.6	59.0	59.6	55.0

* Rate per 100 hundred inhabitants; **NTP-National tuberculosis program; ***DOT-directly observed treatment.

**Table 3:** Factors associated with a short time (≤ 3 months) to full implementation of the FDC-RHZE regimen in Brazil

**Factors**	**Crude odds ratio**	**95% CI**	**Adjusted odds ratio**	**95% CI**
Small population (< 10,000)				
No	1		1	
Yes	16.84	13.39 – 21.17	25.52	19.09- 34.10
Low population density (<88 inhabitants /km^2^)				
No	1		1	
Yes	5.64	4.68 – 6.80	2.33	1.87 – 2.91
Low level of incidence TB (<50/100,000)				
No	1		1	
Yes	4.21	3.57 – 4.96	8.79	6.75 – 11.45
High coverage DOT* (>80%)				
No	1		1	
Yes	2.30	1.79 – 2.95	1.55	1.14 – 2.12
High laboratory confirmation pulmonary TB (>80%)				
No	1		1	
Yes	2.12	1.85 – 2.42	1.93	1.63 – 2.29
High laboratory monitoring (>80%)				
No	1		1	
Yes	1.45	1.27 – 1.67	0.92	0.78 – 1.09
Less than 120 patients treated				
No	1		1	
Yes	134.26	18.73 – 962.41	10.07	1.36 – 74.52

* DOT- Directly observed treatment.

Operational health service performance characteristics, such as more laboratory confirmation and monitoring of cases, were associated with a more rapid implementation. However, the inverse relationship occurs for the proportion of cases under DOT (Table 2). In the multivariate context, the association between speed and laboratory monitoring has lost statistical significance (Table 3). The variable “high laboratory monitoring (>80%)” was not associated in the multiple regression analysis. Thus, it was excluded from the final model.

## Discussion

The understanding of the process of adoption of any new health technology becomes highly important for tuberculosis services as new diagnostic tests [15], treatment regimens [16] and vaccines [17] are expected to emerge in the next few years, after decades of stagnation. How to evaluate these processes, however, is a challenge. Habicht et al. (1999) [18] revised the design options to evaluate public health program performance. They enumerate, as indicators of performance: adequacy, provision, utilization, coverage and impact. The authors also point out that specific interventions should be evaluated considering the initially planned implementation schedule. In the present study, we chose to analyze the time till full coverage of the implementation of a new treatment recommendation in different municipalities in Brazil using secondary data. This was only possible because notification of tuberculosis is mandatory in the country.

Elmahalli et al. (2010) [19] assessed the implementation of DOTS strategy in two chest facilities in Alexandria, Egypt, using treatment success as the outcome. They argue that operational indicators, such as treatment outcome, can be considered excellent tools for monitoring the process of tuberculosis care, especially in developing countries. Although we agree that these are useful indicators of program performance, they are less appropriate to study the implementation process of a new recommendation. In our study, we preferred, instead, to study the implementation process of the new treatment regimen using a different approach, since other relevant data were available in the Brazilian surveillance system.

The implementation of new tuberculosis treatments, including MDR-TB treatment, was reported in different countries [20]. However, these analyses were essentially descriptive. To our knowledge, there are no previous studies addressing factors associated with the implementation process measured as the time to reach full coverage. In the present study, we addressed the factors associated with the time to full coverage of new regimen implementation process. Almost 70% of Brazilian municipalities had notified at least one case of tuberculosis in the study period, which reflects on one hand, the extent of the disease in the country and on the other hand, the expansion of tuberculosis diagnostic activities. Demographic and epidemiological factors as well as operational factors influenced rapid implementation. Among them, demographic factors had the higher magnitude of association. Municipalities characteristics such as population size, demographic density and incidence rates were associated with the rapid implementation of the new regimen. Although in a smaller magnitude, compliance to some NTCP recommendations, such as DOT and microbiological confirmation of cases, also were associated with rapid implementation.

Larger municipalities generally have health systems with many facilities, which need more human resources and health infrastructure, more complex public health program management. They also have populations with a high heterogeneity concerning access to diagnosis and tuberculosis treatment services, which may explain the more rapid implementation in smaller municipalities. This explanation is corroborated by a recent study in Recife, a large city in the Northeast of Brazil, where the incomplete implementation of the epidemiological surveillance management was explained by lack of human resources and incipient performance of planning and Monitoring & Evaluation activities [21]. Likewise, the Family Health Program implementation in Santa Catarina, a wealthy and developed state in Southern Brazil, was firstly implanted in areas less assisted, in small or medium size municipalities, where financial incentive to the program induced rapid implementation. Indeed, one or two family health teams offers high coverage rates in municipalities with up to 10,000 inhabitants [22].

Regardless of the population size, demographic density also delayed the implementation of the new treatment. Poor social condition found in situations of intense urban agglomerations possibly hamper access to health services. It is intuitive to expect that municipalities with small populations or a small number of cases have easier access to new regimens. Areas with large number of cases demand more health units and higher number of trained professionals. On the other hand, higher incidence rates are usually found in situations of worse health policy performance. Lack of professionals, transport problems and insufficient resources for technical assistance were considered the major barriers for tuberculosis control in São José do Rio Preto, a medium size municipality [22].

In the present study, operational indicators such as DOT coverage and microbiological confirmation of diagnosis, as recommended by the NTCP guidelines, were independently associated to rapid implementation of the new regimen. This may reflect willingness, commitment and capacity of municipal TCP in lining-up with new recommendations from the NTCP. None of those different operational aspects represent by themselves a construct regarding the quality of tuberculosis control actions practiced at the municipal level. However, the independent association of those factors indicates that they evaluate different aspects of the same commitment.

Other factors possibly associated with rapid implementation of the new regimen, mainly those related to health service organization (human resources, decentralization of care, municipal tuberculosis control program structure among others), were not considered due to the nature of this study, which used secondary data from the tuberculosis surveillance system. The quality of this database may have influenced the results of this study. Thus, a previous evaluation was done to ensure the quality of the data [7]. Other possible limitations of this study may result from classification of individuals regarding the use of the new regimen, which was based on the use of 4 drugs in new cases, instead of the use of FDCs, since the latter information is not available. This may have overestimated the coverage of the new regimen, but is not likely to have influenced the evaluation of the implementation speed.

## Conclusions

The analysis of the Brazilian surveillance secondary database made possible the evaluation of the implementation process of a new tuberculosis treatment regimen in this continental-sized country, measured by the length of time to reach full coverage. This analytical approach is useful to learn lessons. It was possible to demonstrate that demographic and operational characteristics of municipalities have influenced the implementation process. Municipalities with larger population size, higher demographic density and higher incidence rates may need more attention for the implementation of future new guidelines for tuberculosis control as well as for other diseases. Effectiveness studies are necessary and may be performed using the same methodology.

## Competing interests

The authors declare they have no competing interests.

## Authors’ contributions

JUB conceived the study, analysed data and drafted the manuscript. DAC analysed data and helped drafting the manuscript. AT analysed data and drafted the manuscript. All authors read and approved the final manuscript.

## Pre-publication history

The pre-publication history for this paper can be accessed here:

http://www.biomedcentral.com/1471-2458/13/321/prepub

## References

[B1] Brasil Manual de recomendações para o controle da tuberculose no Brasil 2011 Brasília: Ministério da Saúde

[B2] Gazetta CE Vendramini SH Ruffino-Netto A Oliveira MR Villa TC Descriptive study of the implementation and impact of the directly observed treatment, short-course strategy in the Sao Jose do Rio Preto municipal tuberculosis control program (1998–2003) J Bras Pneumol 2007 33 2 192 198 10.1590/S1806-37132007000200014 17724539

[B3] Gerald LB Bruce F Brooks CM Brook N Kimerling ME Windsor RA Standardizing contact investigation protocols Int J Tuberc Lung Dis 2003 7 12 Suppl 3 S369 S374 14677825 PMC1609960

[B4] BRASIL, Saude Md. Apresentação PNCT 2011 [cited 2012 July 18 2012]; Available from: http://portal.saude.gov.br/portal/arquivos/pdf/apres_padrao_25_01_11_site.pdf

[B5] Brasil Nota técnica sobre as mudanças no tratamento da tuberculose no Brasil para adultos e adolescentes. Norma técnica 2009 Brasília: Ministério da Saúde/ Secretaria de Vigilância em Saúde/ Departamento de Vigilância Epidemiologica/ Programa Nacional de Controle da Tuberculose

[B6] WHO Global tuberculosis control: WHO report 2011 2012 Geneve: World Health Organization Report No.: WHO/HTM/TB/2011.16

[B7] Braga J Conceição D Schlussel M Loureiro R Relatório técnico - Estudo da efetividade e da aceitabilidade do tratamento da tuberculose com o esquema 4 em 1 DFC (Dose Fixa Combinada) 2011 CEPESC - UERJ: Rio de Janeiro

[B8] Felisberto E Carvalho EF Maggi RS Samico I Avaliação do processo de implantação da estratégia da Atenção Integrada às doenças prevalentes da infância no Programa Saúde da Família, no estado de Pernambuco, Brasil Cadernos de saude publica / Ministerio da Saude, Fundacao Oswaldo Cruz, Escola Nacional de Saude Publica 2002 18 1737 1745 10.1590/S0102-311X2002000600028 12488901

[B9] Felisberto E Freese E Alves CKA Bezerra LCA Samico I Política de monitoramento e avaliação da atenção básica no Brasil de 2003 a 2006: contextualizando sua implantação e efeitos Rev bras saúde matern infant 2009 9 3 339 357 10.1590/S1519-38292009000300013

[B10] Henrique F Calvo MCM Grau de implantação do Programa Saúde da Família e indicadores sociais Cienc Saude Coletiva 2009 14 sup 1 1359 1365 10.1590/s1413-81232009000800008 19750344

[B11] Nagahama EEI Avaliação da implantação de serviços de saúde reprodutiva no Município de Maringá, Paraná Brasil Cad saúde pública 2009 25 Sup 2 S279 S290 19684935 10.1590/s0102-311x2009001400010

[B12] Quinino LRM Costa JMBS Aguiar LR Wanderley TNG Barbosa CS Avaliação das atividades de rotina do Programa de Controle da Esquistossomose em municípios da Região Metropolitana do Recife, Pernambuco, entre 2003 e 2005 Epidemiologia e Serviços de Saúde 2009 18 4 335 343

[B13] de Sa LD de Andrade MN Nogueira Jde A Villa TC de Figueiredo TM de Queiroga RP Implantacao da estrategia DOTS no controle da Tuberculose na Paraiba: entre o compromisso politico e o envolvimento das equipes do programa saude da familia (1999–2004). [Implementation of the DOTS strategy in the control of TB in Paraiba: between the political commitment and the involvement of the teams of the family health program (1999–2004)] Cien Saude Colet 2011 16 9 3917 3924 10.1590/S1413-81232011001000028 21987335

[B14] StataCorp LP Stata graphics reference manual: release 10 College Station 2007 StataCorp LP: Texas (College Station is a city in Brazos County, Texas)

[B15] Cobelens F van den Hof S Pai M Squire SB Ramsay A Kimerling ME Which new diagnostics for tuberculosis, and when? The Journal of infectious diseases 2012 205 Suppl 2 S191 198 10.1093/infdis/jis188 22476716

[B16] Gardner CA Acharya T Pablos-MÃ©ndez A The global alliance for tuberculosis drug development--accomplishments and future directions 2005 26 2 341 10.1016/j.ccm.2005.02.008 15837115

[B17] Kaufmann SHE Hussey G Lambert PH New vaccines for tuberculosis Lancet 2010 375 9731 2110 2119 10.1016/S0140-6736(10)60393-5 20488515

[B18] Habicht JP Victora CG Vaughan JP Evaluation designs for adequacy, plausibility and probability of public health programme performance and impact Int J Epidemiol 1999 28 1 10 18 10.1093/ije/28.1.10 10195658

[B19] Elmahalli AA Abdel-Aziz BF Assessment of the implementation of DOTS strategy in two chest facilities in Alexandria, Egypt Eastern Mediterranean health journal = La revue de sante de la Mediterranee orientale = al-Majallah al-sihhiyah li-sharq al-mutawassit 2007 13 5 1085 97 18290402 10.26719/2007.13.5.1085

[B20] Furin J Bayona J Becerra M Farmer P Golubkov A Hurtado R Programmatic management of multidrug-resistant tuberculosis: models from three countries Int J Tuberc Lung Dis 2011 15 10 1294 300 10.5588/ijtld.10.0591 21669029

[B21] Bezerra LC Freese E Frias PG Samico I Almeida CK Epidemiological surveillance at the municipal level: evaluation of the degree of implementation Cad Saude Publica 2009 25 4 827 839 10.1590/S0102-311X2009000400014 19347209

[B22] Nogueira JdA Ruffino-Netto A Villa TCS Monroe AA Lucca MES Implantação da estratégia DOTS no controle da tuberculose em Ribeirão Preto, São Paulo (1998-2004). Boletim de Pneumologia Sanitária 2006 14 3 141 144

